# Stakeholder views of the development of a clinical quality registry for interventional radiology: a qualitative study

**DOI:** 10.1186/s12913-021-07423-y

**Published:** 2022-01-09

**Authors:** Ylva Haig, Eli Feiring

**Affiliations:** 1 Department of Radiology, Oslo University Hospital- Ullevål, PO Box 4950 Nydalen, 0424 Oslo, Norway; 2 Department of Health Management and Health Economics, University of Oslo, PO Box 1089 Blindern, 0317 Oslo, Norway

**Keywords:** Clinical quality registry, Qualitative study, Interventional radiology, COM-B, Theoretical Domains Framework

## Abstract

**Background:**

Clinical quality registries (CQRs) can likely improve quality in healthcare and research. However, studies indicate that effective use of CQRs is hindered by lack of engagement and interest among stakeholders, as well as factors related to organisational context, registry design and data quality. To fulfil the potential of CQRs, more knowledge on stakeholders’ perceptions of the factors that will facilitate or hamper the development of CQRs is essential to the more appropriate targeting of registry implementation and the subsequent use of the data. The primary aim of this study was to examine factors that can potentially affect the development of a national CQR for interventional radiology in Norway from the perspective of stakeholders. Furthermore, we wanted to identify the intervention functions likely to enable CQR development. Only one such registry, located in Sweden, has been established. To provide a broader context for the Norwegian study, we also sought to investigate experiences with the development of this registry.

**Methods:**

A qualitative study of ten Norwegian radiologists and radiographers using focus groups was conducted, and an in-depth interview with the initiator of the Swedish registry was carried out. Questions were based on the Capability, Opportunity and Motivation for Behaviour Model and the Theoretical Domains Framework. The participants’ responses were categorised into predefined themes using a deductive process of thematic analysis.

**Results:**

Knowledge of the rationale used in establishing a CQR, beliefs about the beneficial consequences of a registry for quality improvement and research and an opportunity to learn from a well-developed registry were perceived by the participants as factors facilitating CQR development. The study further identified a range of development barriers related to environmental and resource factors (e.g., a lack of organisational support, time) and individuallevel factors (e.g., role boundaries, resistance to change), as well as several intervention functions likely to be appropriate in targeting these barriers.

**Conclusion:**

This study provides a deeper understanding of factors that may be involved in the behaviour of stakeholders regarding the development of a CQR. The findings may assist in designing, implementing and evaluating a methodologically rigorous CQR intervention.

**Supplementary Information:**

The online version contains supplementary material available at 10.1186/s12913-021-07423-y.

## Background

Clinical quality registries (CQRs) collect individual-based observational data about medical interventions, procedures, service delivery and outcomes in a specific area of healthcare. They provide an important source of information on the development and monitoring of care quality, variations in best practice treatments and outcomes across centres, adherence to and development of clinical practice guidelines, and performance, as well as for clinical and health service research [1–10].

Previous studies suggest that a CQR may contribute to quality improvement and opportunities for research [11–13]; however, the underuse of clinical registry data in quality improvement and for research purposes has been suggested in earlier studies [3, 9, 14–16]. While the characteristics of the registry itself, such as the registry design, the procedures for data registering and governance, data quality and relevance and the possibilities for feedback, seem to be important determinants of use, additional factors related to organisational context, including management support, and individual level factors, such as a lack of skills, engagement and interest on the part of stakeholders, are considered further barriers to the effective usage of registry data [10, 16, 17].

It is well known from the implementation literature that genuine commitment and support from the parties involved is needed for intervention use. Involving stakeholders in all phases of the process is instrumental to this end [18]. Accordingly, more knowledge about stakeholder perceptions of CQRs as a tool for quality improvement, including opportunities for research, is needed to achieve implementation objectives related to the development and subsequent use of CQRs.

To contribute to the research on the development phase of CQRs, and more specifically, gain a better understanding of the potential determinants of establishing a national clinical quality registry for interventional radiology (CQR-IR) in Norway, we aimed to explore stakeholders’ views on which factors they perceived to be facilitators of or barriers to developing a national CQR-IR, as well as to identify intervention functions likely to enable CQR development.

Interventional radiology involves minimally invasive imageguided therapeutic procedures, as well as invasive diagnostic imaging. We believe this clinical field serves as an interesting case study because it is one of the most technologically advanced medical specialties and undergoing exponential development in term of new techniques and devices. Implementing a national CQR may contribute to developing the knowledgebase of interventional radiology further because a registry can enable the collection, accessing and sharing of important outcome data; evidence-based medicine and improved patient care.

As far as we know, there is only one national CQR-IR in the world, the recently established Swedish Registry in Interventional Radiology (SRIR). To provide a broader context for the present study on the determinants perceived by radiologists and radiographers to affect CQR-IR development, we additionally sought to include experiences from the development phase of the SRIR.

## Methods

### Design and setting

This study was a prospective qualitative study using semi-structured interview and focus groups. The research was theory-driven, and the theoretical framework is described below. The COREQ checklist was used in reporting the study.

Participants were recruited from two countries, Norway and Sweden. Norway (approximately 5,5 million people) and Sweden (approximately 10 million people) have universal tax-based healthcare systems, comprehensive systems of personal identification numbers and high level of generalisable trust, all of which are conditions supposed to favour the development and implementation of CQRs [19]. National registries are organised within a central governance structure and regulative framework. National CQRs receive government funding in both countries. Table 1 shows an overview of the aims and governance of CQRs in Norway and Sweden.

**Table 1:** Aims and governance of national Clinical Quality Registries (CQRs) in Norway and Sweden

Aims	•Quality improvement •Decision support •Research
Governance	•Governmentally funded •Regulated by law •Self-governance •Favorable patient data regulation •Publicly funded competence centres •Patients may opt-out •Norway: providers required to report data •Sweden: providers not required to report data

In Norway, 52 national CQRs are established to safeguard the quality of care via statistics, analyses and research. In addition, personal health data can be used for planning, governance and preparedness purposes by healthcare services and authorities. Healthcare professionals may initiate registry development but the registry must be further supported by the relevant professionals. The four regional health authorities are responsible for establishing, organising and financing the national CQRs. An expert group is consulted when a registry applies to be given status as a national registry, and the regional health authorities jointly handle the application. The Directorate of Health and Care Services is responsible for the final decision based on the evaluation of four criteria; (i) a lack of consensus on diagnostics and treatment, (ii) the potential for quality improvement, (iii) expected losses of prognosis due to a lack of knowledge, and (iv) patient groups or treatment options in which the relative cost–benefit ratio is not well documented. A competence centre for the national CQRs has recently been established, promoting the development of new registries and facilitating collaboration between registries. Norwegian healthcare providers are obliged to report to national CQRs [1]. Also, CQRs are required to use standardised variables and national standards registries. There, personal data may be collected and used without consent; however, the patients must be informed regarding registration. Patients may opt-out of the registry, and their data will be deleted. Before data can be used for a specific research project, approval must be obtained from an ethics review board, and data from different registers may be merged into a de-identified data-base after ethical approval.

In Sweden, 109 national CQRs are established to improve quality of care, research and benchmarking. Health professionals have initiated most of these registries. Registries are fostered by favorable patient data regulation and cofounding by the government and local authorities, soft regulation and professional self-governance [19]. It is the responsibility of the health professionals to initiate, design and manage national registries. Each registry has a steering group, a registry holder and a registry management and a county council is legally responsible. The CQRs are divided into four levels of certification, which are used as a means of controlling funding. Six regional quality registry centres support the national registries. Swedish healthcare providers are not obliged to report to national CQRs. However, the required datasets are succinct and participation rates are high. Patients must be informed that their data will be recorded, but they have a right to opt-out of the registry. Before data can be used for a specific research project, approval must be obtained from an ethics review board, and data from different registries may be merged into a de-identified database after ethical approval.

### Theoretical framework

A growing body of literature supports the use of theory-based research on the development and implementation of healthcare interventions [20]. Theory aids in unpacking the complex relationship between context, content, application and outcomes and, thus, enables a situational understanding of the effectiveness of the intervention [21]. For the purposes of this study, we used a theoretical framework, COM-B, to identify factors perceived by radiologists and radiographers to affect CQR-IR development and, further, to identify intervention functions that can subsequently contribute to a theoretically informed implementation of such a registry.

**Fig. 1:**
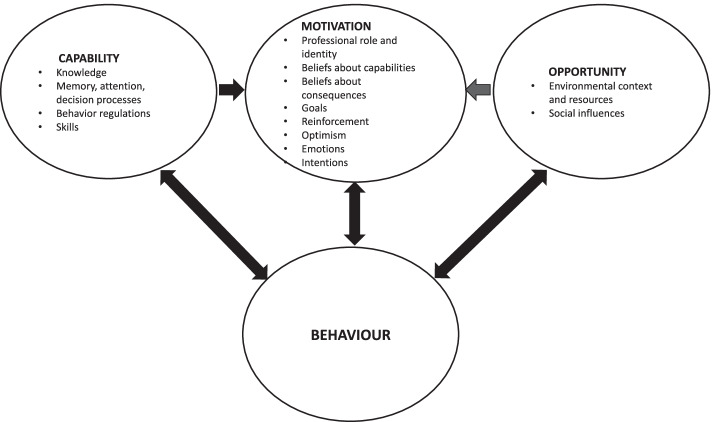
The relationships between COM-B components and the TDF domains, adapted from [22, 24, 25]

The analytical model COM-B enables a theoretical understanding of how capability (C), opportunity (O) and motivation (M) interact to generate behaviour (B) [22]. In short, capability is the individual’s psychological and physical capacity to engage in an activity. Motivation is the reflective and automatic processes that direct behaviour. Opportunity is the physical and cultural-social factors such as environmental, organisational, and social context and resources, that lie outside the individual and make behaviour possible. These three components can influence one another in various ways. We used the Theoretical Domains Framework (TDF) to conceptualise sub-themes of the three main behavioural components. The TDF (version 2) categorises 14 domains relevant to behaviour [23]. The relationships between the COM-B components and the TDF domains are illustrated in Fig. 1.

The COM-B Model has been further developed to help identify potentially relevant intervention functions aimed at addressing deficiencies in the salient COM-B components [22]. Nine intervention functions are specified in a Behavioural Change Wheel (BCW): education, persuasion, incentivisation, coercion, training, restriction, environmental restructuring, modelling and enablement. Potential interventions should target the likely determinants of behaviour to increase the likelihood of an intervention to being successful. For example, capabilities can be achieved through education, training and enablement, while motivation can be achieved through increasing knowledge and understanding, incentivising behaviour or restricting it, and eliciting positive or negative feelings via persuasion or coercion. Opportunities can be achieved through enablement and environmental change, as well as restrictions.

### Sampling and recruitment

We wanted to include participants who would provide theoretically meaningful data and accordingly the study participants were selected purposively [26]. We contacted the head of the interventional radiology department at a large university hospital in Norway. Potential participants consisting of interventional radiologists and radiographers were informed about the study and invited to focus group discussions. Furthermore, we approached the initiator of the Swedish SRIR and asked for an individual interview. The recruitment process benefited from the clinical knowledge of the research team (YH being an interventional radiologist) and pre-existing relationships with the Norwegian clinical team and the Swedish participant.

### Data collection

A semi-structured interview guide was developed, with open questions about perceptions of stakeholders’ capabilities and motivation to develop a CQR-IR, as well as their organisational opportunities in relation to establishing a registry (Supplementary File 1).

Two focus groups were arranged (one with radiologists and one with radiographers). They took place in August 2019 during working hours and lasted about 45—60 min. The Swedish participant was interviewed by telephone in September of 2019. All data were collected by one of the authors (YH). Data were audio-taped on a password-protected smartphone, uploaded to a university server-connected computer, and subsequently transcribed verbatim by the interviewer.

### Data analysis

Data were analysed thematically [26]. To aid in the analyses, we used the COM-B Model [22], combined with the Theoretical Domains Framework [23, 24].

First, we coded and organised data in accordance with the COM-B Model. Then, fragments of the text were categorised and then re-categorised into sub-themes that were comprehended as relevant to the study according to the TDF. Both authors participated in the data coding. In cases of discrepancies in interpretation, these were discussed among the authors until agreement was reached. Illustrative quotes were selected to demonstrate the various themes and sub-themes and translated into English by one of the authors (YH). All participants were given the opportunity to read copies of the data transcripts and provide feedback.

### Ethics

The research project was notified to the hospital and the Norwegian Centre for Research Data (Project number 505265) and found to be in accordance with data protection legislation. Following the initial approach, the participants were provided with written information about the study, including the information that participation was voluntary. Participants gave their informed consent to participate. They were presented with the citations and approved the use of citations in the article. All data were kept on a password-protected computer and only accessible by the authors.

## Results

Five interventional radiologists (denoted 1—5) and five radiographers (denoted A—E), all working at the radiology department at a large Norwegian urban university hospital, were informed about the study and invited to participate. None declined the invitation. Furthermore, the initiator of the SRIR (denoted X) was included in the study. The characteristics of the participants are presented in Supplementary File 2.

We found that the barriers to and facilitators of the development of a CQR-IR, as perceived by the stakeholders, were related to the three behavioral components, which were categorised into eight theoretical domains: Capability (knowledge); Motivation (goals, beliefs about consequences, professional role, beliefs about capabilities, emotions); and Opportunity (resources, social influences). Domains that were not found to be relevant included skills, decision processes, and behavioral regulation.

### Capability

Participants identified knowledge about the rationale behind implementing a CQR as an important determinant of CQR-IR development. Also, CQRs were acknowledged as a source of quality, quality improvement and research. Participants also stated that knowledge about how to implement a CQR, and in particular a CQR-IR, would facilitate such development. For example, the radiologist group discussed the way in which the Swedish experience with establishing a CQR-IR could serve as a learning case.

*It seems like an incredible lot of work to do it all from scratch. Therefore, if someone else has done something similar it seems very tempting (to learn from him or her).* (1)

The Swedish interviewee likewise highlighted the importance of learning from prior experience about CQR development.

*I had a gateway to someone who knew what it was all about and knew how to implement registries, so he helped me (…). You can learn a lot from us. Developing a registry (…) is very time consuming and we can help each other. The parameters are likely very similar. We have the same kind of patients and the same diseases, so there is a good reason for cooperating*. (X)

A perceived enabler identified by the radiologists was learning from a pilot project. A smaller registry could facilitate learning before the planning of a comprehensive registry.

*An initial pilot study or a small scale registry would be a good idea*. (4).

### Motivation

The purposes and potential benefits of a CQR-IR were intensely discussed among the participants. Three aims were present across discussions: quality, training and research.

*A registry would be valuable for procedural quality control, research and (the) training of new staff*. (C).

The radiologist group further believed that the development of a CQR-IR should aim for national status.

*The goal with such a registry should be that others adopted it and made an equivalent registry*. (2)

Overall, there was a common thought that the development of a registry would have to be perceived as beneficial by stakeholders.

*I think we struggle to do new things we don´t see an immediate effect of*. (C).

Participants believed that the establishment of a CQR-IR would have a range of favorable consequences and in effect, have the potential to be useful. They agreed that there was room for the improvement of procedures and routines. For example, the radiologists discussed how the registry should enable more efficient registration procedures and reporting. A perceived enabler of the development of a CQR-IR identified by the radiologists was “*manageable*” data registration and data being linked directly to a radiology report.

*One should aim at integrating the registry in the existing radiology information system so that a systematic report of the interventional procedure is created simultaneously… You enter the data, and the outcome is a report. It is crucial that the registry facilitates a systematic radiology report to avoid double work efforts.* (2)

Participants also thought that the establishment of a registry would make research data more accessible. The potential for research was identified as an enabling factor.

*An IR registry is fun if you get good reports and additionally it´s nice if you get interesting data that can serve as a basis for research*. (X)

Radiographers expressed the need to replace the existing workflow, in which they register procedural data on the computer and, in addition, fill out checklists on paper later stored in folders. They believed that the development of a registry would enable digitalising the registration of the IR procedures, avoiding the unnecessary duplication of data entry and lightening the workload. Furthermore, they believed that a registry would facilitate better control of equipment use and stock in the angio suite. This was thought to help in ordering the correct amount of new and sometimes very expensive equipment with a limited expiry date, potentially saving both time and money.

A major barrier to the development of a CQR-IR identified by the radiographers was the professional role and identity of the radiologists. They felt that elements of the radiologist role, such as a clinical rather than an administrative focus, were a cause of concern and that it could be difficult motivating some of the radiologists to complete registry data. In effect, the registration process should be simple.

*Doctors find all kinds of excuses for not doing it (data-registering)*. (1).

The radiologists discussed the professional role more indirectly and stated that a major barrier was agreeing with colleagues on the design of and content in the registry. They stressed the importance of limiting the dimensions of the registry to facilitate the completion of registry data and consequently its´ success. The user friendliness of the CQR was identified as an enabler.

*Very intuitive variables and easy registration (are necessary) to make it feel worthwhile. I believe it will be more difficult (…) if it is a very comprehensive registry so it has to be user friendly*. (A)

Across the focus groups and interview, the participants identified a positive and enthusiastic organisational culture as an enabler of the development of a CQR.

*The most important thing is getting the colleagues to cooperate. To create some kind of enthusiasm (…) You need to find a few enthusiasts you get along with and can work with.* (X)

The participants stated that it was important to work together with motivated people and create enthusiasm around the project.

*Not everybody should be involved and especially not those who are not interested*. (1).

Negative emotions were identified as a major barrier. Such emotions included stress, change resistance and work fatigue. Some of the participants talked about difficult experiences from previous change processes. In addition, a certain lack of optimism and willingness to change correlating with age was described.

*Some doctors are just tired (…). The older colleagues are used to the existing system and may have difficulties adapting to new systems*. (X)

Furthermore, participants reported that an expectation of quality improvement and a greater focus on quality indicators made them feel uncomfortable because of not being able to answer questions about outcomes. A fear of complaints because of not having registered data and embarrassment due to not knowing about the complications associated with implants, were discussed among the radiologists. They felt that these negative emotions could potentially become a motivator for change and development.

*We are required to get an overview of the quality of our procedures. We don´t have that quality control of our activity, which is unacceptable (…) it certainly is (…) in 2019, with the transparency and the requirements to show your own data (…). It would be incredibly embarrassing if someone came and asked: How many Viatorr (implants) have you inserted in your TIPS patients and have you had any complications related to them? (…) We could only speculate. We don´t have any data.* (1)

### Opportunity

Participants highlighted the importance of organising a steering group to achieve a satisfying progress in the development process. Some argued that a few motivated and resourceful members should constitute the group. Having an active role in the steering group seemed to persuade others in the discussion to overcome much of their skepticism.

*A selected group of interventional radiographers could ensure the continuity.* (4).

Some were critical of such a strategy, however. They believed that the registry would rise and fall along with the enthusiasts.

*It falls apart after a few years. When the enthusiast is gone the database also disappears*. (2).

Participants identified a lack of designated resources and cost constrains as potential barriers to the development of a CQR. They perceived financial resources as an important determinant of development. Some of the participants argued that the implementation of a CQR would be costly and difficult to prioritise. Others thought that a CQR would lead to better control of the stock of equipment and expensive implants, thus saving money.

A lack of time to register data was another barrier to the development of a CQR, as perceived by the participants. In particular, the radiographers felt that time was already stretched due to other new working tasks, and that the implementation of a CQR-IR would imply an additional workload on them. Having designated time in which to register data would facilitate registry implementation.

*When you are busy on-call or at night, you don´t want to sit and register retrospectively*. (B).

The participants believed that leadership support was essential to the successful development of a CQR and a motivating factor in itself. The Swedish interviewee supported these views.

*You can´t manage it all yourself (…). You should have support from your own leaders, backing you up*. (X)

Nevertheless, participants reported that management did not necessarily prioritise quality improvement initiatives.

*Some of the leaders are concerned about quality improvement (...). They are very positive when you talk to them but become silent when you ask for support. Then it gets difficult*. (A)

In addition to leadership support from one’s own organisation, support from “the mother association”, such as the Radiology and Interventional Radiology Association, was identified as a facilitator. The Swedish interviewee underscored this point.

*I was meticulous about having support from the associations*. (X).

## Discussion

The demands and expectations in society regarding quality, quality improvement and patient safety have led to health systems´ increased focus on the documentation of procedures, results and complications. A functioning CQR is often considered a prerequisite for quality improvement, as an aid to identifying and improving health outcomes [9, 11, 12]. The development and implementation of change processes, such as a new registry, go through a number of steps, from the initial start-up to the continuous use of data. Recent developments in implementation theory encourage a systematic approach to the identification of facilitators of and barriers to all phases of an implementation process [20]. Various factors determine the success of a change process, including the organisational and structural conditions of its implementation, the resources available, and factors connected to the professionals that will carry out the intervention [18]. Using the COM-B and TDF, our study has identified enablers of and barriers to the development of a CQR-IR, as perceived by stakeholders.

Enablers included knowledge about the rationale for establishing a CQR-IR and beliefs about the beneficial consequences of establishing a registry. Quality is a central aspect of healthcare delivery, and the participants agreed on the need for quality documentation and the importance of quality improvement in the quickly developing and technically advanced field of interventional radiology. The participants thought that a registry would improve the quality of work and ease the total work burden by providing a systematic radiology report, improve the control of the usage and stocking of equipment and, further, facilitate research.

In addition, participants felt that support from the wider interventional radiology environment was an important facilitator of registry development. The possibility of learning from the experiences of a well-developed CQR-IR, namely the SRIR, was also perceived as a facilitator. These findings align with research that has underscored collegial calls for local results, as well as the interest and engagement of team members and managers, as important factors for the use of a registry [16].

The present study identified change resistance as the most important barrier to registry development. Role boundaries were perceived as hindering the potential implementation of a CQR-IR, as explained by the differing professional identities of radiologists and radiographers. Across groups, resistance to a potential implementation process and further, the potential managing of a registry was reported. Negative emotions seemed to be triggered by prior experiences with continuous organisational change, work fatigue, and stress.

Previous research has underscored how developing and establishing a new registry requires a long-term commitment of resources, as well as change in infrastructure and the wider organisation [8]. The participants in our study identified a range of organisational factors perceived to hinder registry development, including lack of designated resources, such as staff, financial resources and time, as well as a lack of leadership support.

The results of our study highlight the need for strategies that are likely to be effective in responding to the concerns of the radiologists and radiographers about their capabilities, opportunities and motivation to develop a CQR-IR when faced with a heavy workload, change resistance and resource constraints.

The Behavior Change Wheel (BCW) described above provides a systematic and theory-guided approach to identifying the intervention functions most likely to achieve change in a particular behavior [22]. Any given intervention can involve more than one intervention function. Thus, we can use the BCW to design interventions that incorporate several intervention functions likely to achieve change, given the constraints perceived by the participants. Implementing changes is a stepwise process and different types of interventions may be needed in the different steps. Establishing a pilot study may be appropriate in the early phases of the process, and most registries initially start as a pilot study [8]. A pilot study is favorable because it is a small-scale activity that can be finished and evaluated within a short time. Moreover, previous implementation studies of CQRs have underscored the importance of establishing a formal procedure with support from management and local champions or registry advocates. A pilot study provides an opportunity to enact new procedures.

A pilot study can incorporate a range of intervention functions. It requires *environmental restructuring*, given that resources will need to be allocated to the project, and formalising local clinical leadership who are accountable for ensuring registry development and subsequent implementation. Furthermore, a formal steering group should be established, possibly with representation on the part of associations, a competence center and well-developed CQRs.

Additionally, *persuasion* and the *incentivisation* of stakeholder participation will facilitate *education* that focuses on the development and subsequent implementation of a CQR. A pilot study would further facilitate appropriate *training* to develop the necessary skills with which to design a user-friendly registry and make use of the data for various purposes.

Previous research into quality improvement initiatives suggest that both clinical and managerial leadership are needed to bring about new ways of working [27]. In terms of motivation to develop a CQR-IR, the participants in the present study identified role conflicts as a potential barrier to developing such a registry and highlighted the importance of good clinical leadership in fostering collaboration between radiologists and radiographers. Thus, *enabling* teamwork involving the different groups and mutual engagement in the development process can reduce barriers to increased participant capability and motivation.

Likewise, leadership is important in developing an understanding of the need for organisational mechanisms that help improve healthcare quality and safety. Sustained changes in the motivation to develop and subsequently use a CQR may involve the development of an organisational and social culture that value evidence-based practice.

Suggested intervention functions to change behavior so as to facilitate the implementation of the registry are presented in Table 2.

**Table 2:** Determinants of CQR-IR development identified in focus groups and interview

COM-B components	TDF domains	Specific factors identified as determinants for CQR-IR development	Potential intervention functions
Capability	Knowledge	Knowledge about CQR rationale	Education about CQR
		Knowledge about implementing CQR	Training in registry development and use
Motivation	Goals	Aims of CQR	Education about CQR
	Beliefs about consequences	Benefits and costs	Participant persuasion
	Professional role	Role boundaries of radiologists and radiographers	Participant persuation and incentivising
	Beliefs about capabilities	Registry user-friendliness	Participant incentivising
	Optimism	Organisational culture	Enabling teamwork and mutual engagement
	Emotions	Resistance, stress	Enabling teamwork and mutual engagement
Opportunity	Resources	Staff, financing, time, management support	Environmental restructuring
	Social influences	Associations	
		SRIR	

### Strengths and limitations

This study utilised the theoretical COM-B Model and TDF as part of the Behavior Change Wheel [22, 24]. We consider the theoretical basis of the study to be a true strength because it enables comparisons with other studies. However, the predetermined analytical framework may have led us to overlook important factors. Moreover, coding was sometimes difficult because data were ambiguous and text units seemed to fit into multiple domains. We coded the text units into the domain that we deemed to best reflect the key theme, as suggested by the developers of the COM-B/TDF framework [24].

The study used data from two countries, adding valuable insights beyond the single registry. Because interventional radiology is a small and highly specialised field within medicine, the sample was small and the participants were recruited from only one hospital in Norway, in addition to the Swedish interviewee. However, we strove to include both radiologists and radiographers, with different roles and experiences, in the study. Because we used a deductive approach, we did not aim for saturation understood as a criterion for discontinuing data collection. Rather, we aimed for an adequate representation of the predetermined categories in the data (deductive thematic saturation) [28, 29].

Broad and open-ended questions were used with the intention of making participants discuss the topics freely in the focus group and provide reflections in the individual interview. Despite informing the participants at the beginning of each focus group of the aim of the study and the investigator’s role in the present setting, we cannot disregard the interaction between the investigator, who is an interventional radiologist, and the participants. Furthermore, we are well aware of the fact that focus groups may increase the conformity of responses. We cannot disregard potential interaction between the participants, which may have affected their views, with participants potentially mirroring one another opinions. In addition, because we recruited groups of knowledgeable participants, the viewpoints the participants discussed may differ from those of the local or wider workforce.

The findings have limited generalisability, yet the study’s theoretically informed analysis allows the transferability of insights to other settings, e.g., the implementation of other CQRs in interventional radiology or other medical specialities. The analytical framework makes it possible to systematically compare our results with those of other studies and across fields. Further research is needed to confirm reproducibility and transferability in other settings and thus answer questions concerning how far this framework can be used in the design of specific and effective interventions. Conducting a similar study on a larger scale, for example by interviewing interventional radiologists and radiographers in Sweden, the only country to have established a national CQR-IR, or incorporating quantitative methodologies may provide a better understanding of the issues discussed in this present study. Future studies may further include comparing the development process of CQRs in different medical fields.

## Conclusion

Developing a CQR-IR, potentially with the aim of implementing a national Norwegian registry, will require organizational changes and must be backed up by a demonstrated potential for quality improvement and research. Registry development may be challenging in a hectic and demanding work place. This study’s results may be important in determining what must be changed if such a registry is to be created and which intervention functions are likely to bring about the needed changes.

## Supplementary Information

**Additional file 1.** Demographic details.

**Additional file 2.** Topic guides: Focus groups and interview.

## Data Availability

The data were originally collected for the purpose of a Master’s thesis in Healthcare Administration and are available from the corresponding author upon reasonable request.

## Abbreviations

COM-B: The Capability, Opportunity, and Motivation Behaviour Model
TDF: The Theoretical Domains Framework
CQR: Clinical Quality Registry
CQR-IR: Clinical Quality Registry – Interventional Radiology
SRIR: Svenskt Register för Interventionell Radiologi («Swedish Registry for Interventional Radiology»
