# Supplementary material for: Stakeholder views of the development of a clinical quality registry for interventional radiology: a qualitative study
Source: BMC Health Serv Res. 2022 Jan 9;22:44. doi: 10.1186/s12913-021-07423-y (PMC8742914; doi:10.1186/s12913-021-07423-y)
Supplement: Supplementary file 1 — Additional file 1. Demographic details. [file 12913_2021_7423_MOESM1_ESM.docx]

Demographic details

| **Gender** | **Age** | **Education** | **Experience in IR** |
| --- | --- | --- | --- |
| Male | 59 | Radiologist | >20 years |
| Male | 65 | Radiologist | >30 years |
| Male | 44 | Radiologist | >10 years |
| Female | 50 | Radiologist | 5-10 years |
| Male | 41 | Radiologist | 5-10 years |
| Male | 33 | Radiographer | <5 years |
| Female | 34 | Radiographer | <5 years |
| Male | 31 | Radiographer | 5-10 years |
| Male | 24 | Radiographer | <5 years |
| Male | 67 | Radiologist | >30 years |
