# Supplementary material for: Stakeholder views of the development of a clinical quality registry for interventional radiology: a qualitative study
Source: BMC Health Serv Res. 2022 Jan 9;22:44. doi: 10.1186/s12913-021-07423-y (PMC8742914; doi:10.1186/s12913-021-07423-y)
Supplement: Supplementary file 2 — Additional file 2. Topic guides: Focus groups and interview. [file 12913_2021_7423_MOESM2_ESM.docx]

Topic guides: Focus groups and interview

| Topic | Focus groups | Interview |
| --- | --- | --- |
| Capability | - What is your understanding of the aim of this registry? - Do you think that certain skills are needed to develop this registry? - Do you think that certain skills are needed to use such a registry? | - What is your understanding of the aim of a CQR-IR? - Do you think that certain skills are needed to develop such a registry? - Do you think that certain skills are needed to use such a registry? |
| Opportunities | - What would the different groups working here think about developing such a registry? - Do you think that organisational resources are needed to develop a registry? - To what extent do you think that the department is equipped to take on such a task? - How should a registry be designed to be useful? - Do you think you can learn from experiences elsewhere? | - Did you experience different attitudes towards establishing a CQR-IR from different professional groups? - Are certain organisational resources needed to develop a registry? - How should a registry be designed to be useful? - Could others learn from your experience? |
| Motivation | - What do you think would be the consequences of establishing a registry? - To what extent do you think that such a registry would be beneficial? - To what extent do you think you have the skills to develop a registry and subsequently make use of it? - Do you think a registry would be a good idea? | - What have been the consequences of establishing a registry? - How is it possible to counteract lack of motivation? |
